# Empathic Narrative of Online Political Communication

**DOI:** 10.3389/fpsyg.2022.869496

**Published:** 2022-04-05

**Authors:** Yuqi Wang, Lihong Lu, Zhibo Zhou, Jing Zhu

**Affiliations:** ^1^School of Humanities, Jiangxi University of Finance and Economics, Nanchang, China; ^2^College of Modern Economics and Management, Jiangxi University of Finance and Economics, Nanchang, China; ^3^School of Business Administration, Guangdong University of Finance and Economics, Guangzhou, China

**Keywords:** online political communication, political culture, empathic narrative, cultural interaction, empathic communication

## Abstract

With the rapid development of the Internet, political culture plays an increasingly prominent role in ethical guidance and value orientation, and the intergenerational inheritance of political culture in various countries needs to be carried out in a sophisticated way. From the perspective of empathic narrative, this study applies the network text analysis method to detect the cultural communication regularities to the contemporary young adults in online political communication and explores contemporary young adults’ perception of online political culture through empirical analysis. Moreover, it proposes the empathic narrative logic of online political communication by comparing the existing communication elements and the urgently needed communication elements. Our findings suggest that we need to deepen the cognitive empathy, stabilize the emotional empathy, and adjust the emotional bias in online political communication.

## Introduction

Having a long history, political communication is a process of symbolic and mediated information circulation between political actors and objects. It aims to effectively guide value orientation and has a profound impact on the development of modernization. Specifically, political communication is a product of interdisciplinary integration. It not only serves the political direction, but also focuses on the nature of communication. As a result, there are two trends in the defining of the category of political communication, namely, the standard of political science and the standard of communication (Datts et al., 2021). Two major schools have emerged in the academia with regard to the concept of political communication. From the perspective of political science, political communication is defined as a type of basic system function, and its result probably maintains or changes the original political culture (Stieglitz and Dang-Xuan, 2013); and from the perspective of communication, political communication is defined as the manifestation of the role of communication in the political process (Gollust et al., 2020).

With the accelerating globalization and the advent of the era of intelligent interconnection, traditional political communication has been severely challenged, and its living space has been seriously squeezed. At the crucial juncture where opportunities and challenges coexist, it has become a top priority to explore new paths of online political communication. The political culture contained in the online political communication is the treasure of Chinese civilization, and the effect of its communication will be directly related to the implementation and popularization of the country’s major policies and cultural values. As the backbone and core audience of online political communication, the factors influencing the young adults’ willingness to participate in political interaction need to be further explored. It is necessary to build an effective and high-quality political communication force, which will facilitate the contemporary young adults to better absorb the nutrition of excellent political culture and participate in the national political practices.

The selection of subjects in the research on online political communication means the choice of perspectives. With a view to break through the limitations of traditional research perspectives, this study introduces the concept of cultural interaction to analyze the correlation between cultural expression and young adults’ psychology in the online political communication to explore the empathic narrative structure of online political communication and propose the future direction of establishing effective interaction between political culture and young adults. Specifically, this study mainly analyzes and answers the following questions in the current situation of online political communication. *Q1:* How to manifest the narrative process of political culture communication and what regularities its mechanism has; *Q2:* How contemporary young adults’ feedback on political culture and what psychological and emotional responses they have; *Q3:* How to classify the empathic narrative paths of political culture and how to carry out the interaction between political culture and contemporary young adults.

## Literature Review

### Political Communication

The political communication is derived from the fields of psychology, politics, and communication, and its research arose out of scholars’ attention to the role of public opinion and propaganda in the First World War. Based on early modernist psychological theories, there have existed studies on the identity between leaders and followers, laying a solid foundation for persuasion and advocacy in this field. Lippmann (1922) was the first to expound the relationship between media and public opinion, and under his influence, some scholars regarded media as a part of the political governance process (Bennett, 1990; Cook, 1998). In the period of theoretical construction of political communication, Deutsch (1963) constructed the theory of *political communication* based on the ideas of political science, which provided a systematic analysis framework for the operation of government and information transmission, while Epstein et al. (1960) emphasized the role of communication in the operation of political system which is an indispensable functional element; since then, the academia has begun to apply political communication to specific empirical research, which is closely related to political elections in democratic countries. The political science works *The American Voter* (Campbell et al., 1960) is a representative of early studies on voting and opinion formation. At the same time, Lazarsfeld (1944) studied the influence of others, especially opinion leaders, on individual voting behavior in the two-level communication. Political communication theories based on empirical research actively developed, in particular, the agenda-setting theory describes the relationship between the views of the audience and media information (Soroka, 2003), and the media information is the basis for the formation of public opinion and affects the governance effect (McCombs and Zhu, 1995; Gross and Aday, 2003). After that, political communication began to develop from empirical research to pluralistic integration. Swanson and Nimmo (1990) emphasized the important role of critical theory in political communication research, arguing that “positivist behavioral research fails to understand the significance world of political actors” and elaborating on political advertising, political debate, political journalism, relations between government and media, etc.

However, the growth of the Internet has raised concerns about a coherent political agenda and important norms of tolerance in democratic countries may be weakened (Sparks, 2001). Affected by this, the academia began to discuss the relationship between the Internet, the public sphere, and political communication, shifting the focus to the interactive dimension of political communication in the public sphere and analyzing the moderating effect of social structural factors on citizen’s participation (Dahlgren, 2005; Habermas, 2006). The shaping of young adults’ participation in politics by social media focuses more on the relationship between emotion and communication and a deeper contextual understanding of political information (Vraga et al., 2015; Anspach, 2017; Pal, 2017). As young adults are the core targets of online political communication, it is crucial to discuss their characteristics in political communication to build effective and high-quality political communication.

### Empathy and Empathic Narrative

The term *empathy* was first translated from German by British psychologist Edward Titchener in 1909 (Chen, 2018). In the opinion of Rogers (1958), empathy refers to a person’s ability to understand and respond to another person’s experiences. Sinclair et al. (2017) compared sympathy, empathy, and compassion, and pointed out that empathy, on the basis of emotional empathy, adds clear characteristics of being motivated by love, altruistic role, actions, and small supernatural act of kindness of the responders. In other words, the affective component of empathy contains an emotional response to another person, which is the consistency shown in the affective state (Cao et al., 2021; Zhou et al., 2021). The application of empathy to practice shows that “Perceived Psychological Empowerment” (PPE) has a moderating effect in “Service Recovery Awareness” (SRA) and emotional responses (Zhang et al., 2020). Different psychological mechanisms also influence consumers’ behavior through social networks (Wang et al., 2020a,b). Tourists increase the supply of narratives by sharing travel photos and stories (Li and Katsumata, 2020; Liu et al., 2021; Zhang et al., 2022), and affective empathy has a significant mediating effect on the relationship between travel attribution and tourist intentions to online celebrity scenic spots (Yi et al., 2021a).

In extant studies, cultural background is considered to be an important moderator of emotional empathy based on cognitive and affective levels (Feshbach, 1975). Kupetz (2020) regarded empathy as a dynamic that can be presented in different ways according to the environment in which it occurs to reveal the relationship between multicultural differences, empathy transmission, and risk prevention and control behaviors in the context of crisis events (Yi et al., 2021b; Wang et al., 2022). At the same time, approaches that focus on the risk and protective factors that promote or hinder the development of empathy in different cultural groups contribute to a better understanding of how empathy evolves and results as a function of cultural orientations and values (Main et al., 2017). By combining empathy and contextualization, the memory construction and interpretation of historical events and characters are evoked in the form of story strategies to evoke emotional responses (Savenije and De Bruijn, 2017; Crețan et al., 2019; Kidd and Sayner, 2019) and extend the depth and breadth of empathy with emotional story presentation.

## Research Design

This study applies the network text analysis and PLS-SEM empirical analysis methods to explore the communication logic of political culture to contemporary young adults and their cognition of political culture and analyze the effective interactive relationship between political culture and contemporary young adults.

In Study 1, in a view to objectively analyze the communication logic of political culture to contemporary young adults in online political communication, the contents on Sina Weibo (a Chinese microblogging website) are taken as the sample, ROST-CM6 software is used for text analysis, and quantitative and qualitative research paradigms are adopted to more objectively process the large amounts of materials and data representations of the online texts (Luo and Zhai, 2017).

In Study 2, a survey is conducted to the targeted audience, which is the most direct and effective method for the research on contemporary young adults’ cognitive feedback on political culture in online political communication. PLS-SEM is adopted to carry out a small sample analysis under a fixed context to further control the influence of other variables on the communication regularities. In line with the ACME empathy measurement scale designed by Vachon and Lynam (2016), this study puts forward the research hypotheses and constructs a cognitive feedback model of contemporary young adults on political culture in online political communication.

### Study 1: Communication Logic of Political Culture

#### Word Frequency and Semantic Network Analysis

**Figure 1:**
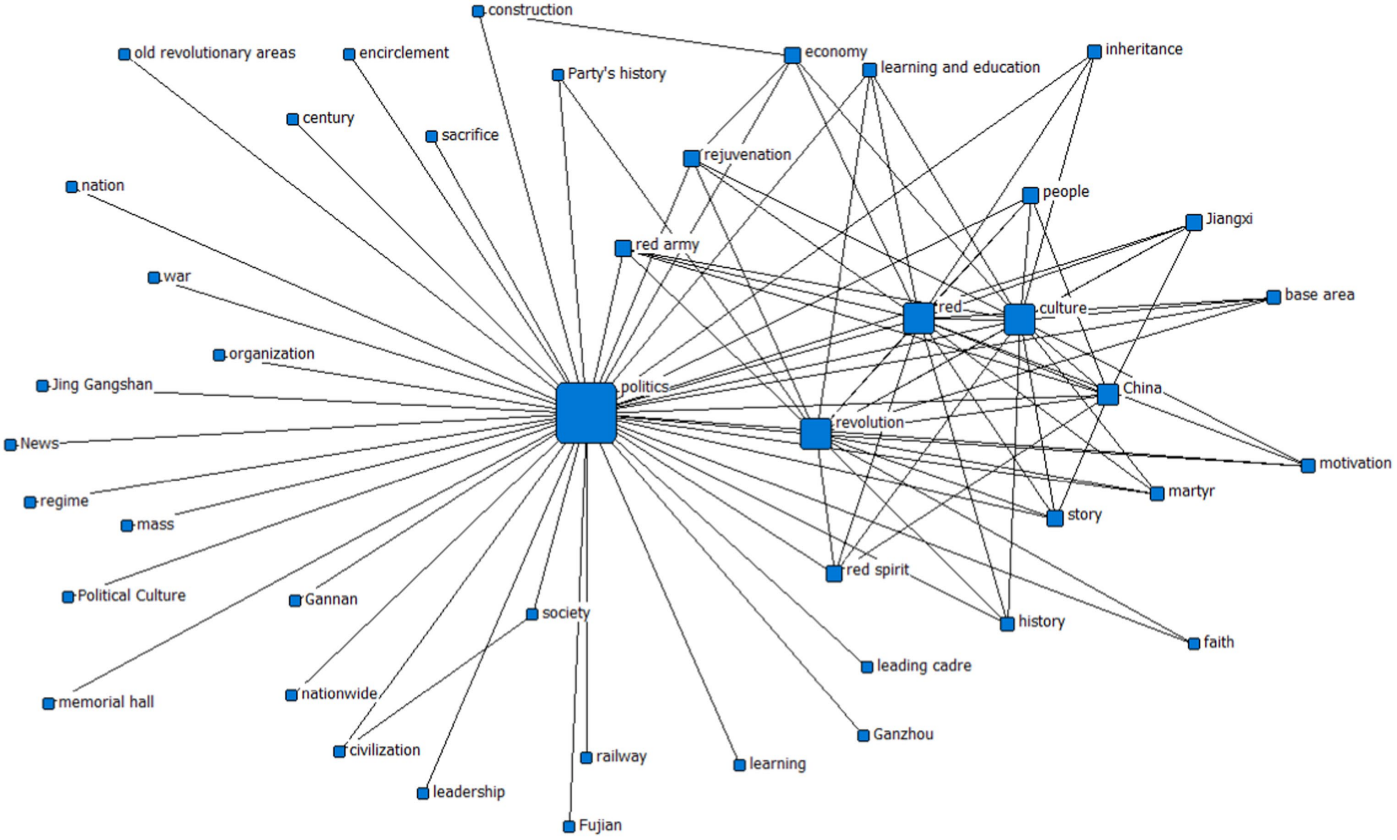
Semantic matrix graph of political culture perception network.

In the word frequency analysis, this study first selected “political culture” as the keyword, and then searched on Sina Weibo to collect and screen the texts. A total of 846 valid posts from 8 January 2018 to May 2021 were collected; second, on the basis of in-depth content cleaning and preprocessing, invalid texts were excluded to clarify the research objects; third, synonyms in the texts were consolidated, such as “Jiangxi” and “Jiangxi province” were classified as “Jiangxi”; and “centenary” and “centennial” were classified into “centennial”; lastly, the texts were imported into ROST-CM6 analysis software, and 44 keywords were finalized as the high-frequency feature vocabulary of 687 samples and their frequencies, among which the top 10 keywords and frequencies are “politics” (1004), “culture” (591), “Red” (533), “China” (502), “rejuvenation” (327), “people” (318), “story” (308), “history” (299), “education” (284), and “civilization” (280). At the same time, the semantic network analysis matrix is drawn (as shown in Figure 1), and the network matrix is found to present the following regularities: “politics” is the core node, “Red,” “culture,” and “China” rank the second, followed by “rejuvenation,” “people,” “story” (story), etc.

#### Coding and the Theoretical Model

Based on word frequency analysis and semantic network analysis matrix, this study conducted grounded theoretical analysis on the Sina Weibo text data for the sake of open coding, spindle coding, and selective coding, and made in-depth analysis of the text contents to construct the cognitive model of the contemporary young adults on the political culture in the online political communication.

#### Open Coding

The study first screened out the political culture contents of the texts on Sina Weibo and encoded them one by one, took the original sentences as the initial concept to the greatest extent, and further integrated the elements with nature similar to the original text contents into categories, for instance, “The reporter came to the second stop of the trip, the birthplace of the red spirit, Ruijin in Jiangxi province,” “There are a large number of exhibits about folk culture in the museum, exquisite and unique folk costume and jewelry, embroidery, silverware, etc.,” “Red stories and ballads circulating here do not fade as the time passes, but shine brighter in the new era,” “The training program lasted for 7 days and carried out various activities, such as young volunteers introducing scenic spots, telling Red Army stories, and red art show and performances. The youngest volunteer was 9 and the oldest was 15 years old.” The following 12 categories were concluded, namely, red historical city, red former architecture, red memorial collection, historical symbol, character symbol, artistic symbol, institutional guarantee, service guarantee, life guarantee, affective identity, belief inheritance, and civilization revitalization.

#### Spindle Coding

Since the categories obtained through open coding are relatively independent and scattered, the relationships among them need to be further explored to make them more conceptual. By analyzing the relationship and logical sequence of the 12 categories, this study finally integrated four main categories which are heritage site carrier, cultural symbol cognition, policy guarantee, and value-oriented education. Specifically, the heritage site carrier includes three categories, such as red historical city, red former architecture, and red memorial collection; the cultural symbol cognition includes historical symbol, character symbol, and artistic symbol; the policy guarantee includes institutional guarantee, service guarantee and life guarantee; and the value-oriented education includes affective identity, belief inheritance, and civilization revitalization.

#### Selective Coding

Through in-depth analysis of the twelve initial categories obtained by open coding and the four main categories obtained by spindle coding, and in line with the theme of this study, the core category was determined to be “political culture,” and its “story line” structure is: historical site carrier—cultural symbol cognition, historical site carrier—cultural symbol cognition—value-oriented education, and cultural symbol cognition—value-oriented education to construct the theoretical model of the political culture communication to contemporary young adults (as shown in Figure 1).

#### Emotional Analysis

On the basis of cognitive analysis of political culture, ROST text software was used to analyze the sentiment of the Weibo texts, including the proportion and intensity of positive, negative, and neutral sentiments, as well as the corresponding comments. It is found that the proportion of positive sentiment of the Sina Weibo users toward political culture was the highest, being 71.90%, and the negative sentiment accounted for only 9.75%. In particular, the highly positive sentiment accounted for the largest proportion, being 27.97%. The analysis of the causes reached the following implications. First, political culture carries the original aspiration and mission of the Chinese people, filled with valuable experience accumulated during the historical struggle; second, political culture is an inexhaustible spiritual driving force for contemporary young adults to bear in mind their original aspirations and missions; third, under the cultural atmosphere and catalysis of learning and education, political culture has been further spread and promoted; fourth, there is cultural loss in the process of intergenerational transmission and contemporary young adults lack the ability to empathize with it.

Along with the continuous deepening and penetration of political and cultural learning and education, the current political culture in online political communication is committed to conveying cultural contents under positive sentiment and has formed a communication mechanism of “historical site carrier → cultural symbol cognition → value-oriented education,” and the policy guarantee adjusts and affects its whole process. However, a crucial question has not yet been deeply analyzed, that is, whether the audience can effectively and rationally perceive political culture. This question directly reflects the effectiveness and problems of current online political communication. Therefore, this study followed the process dimension of “cultural symbol cognition” in the theoretical model of the communication of political culture in the online political communication to contemporary young adults, and sorted out the feedback of contemporary young adults from the perspective of empathic communication, and the result variable cultural identity to further explore the rules of interaction between political culture and contemporary young adults in the online political communication.

### Study 2: Cognitive Feedback on Political Culture

#### Audience Feedback (Empathy) and Cultural Symbol Cognition

Empathy is an emotional response that an individual tries hard to understand another individual (Batson et al., 1981). It includes the bottom-up emotional sharing process (Gazzola et al., 2006) and a top-down cognitive adjustment process (Lawrence et al., 2004), namely, emotional empathy and cognitive empathy. The former is an emotional response to others’ situation and circumstance, and the latter is an understanding of the causes of others’ emotional state. The application of empathy theory is typically scientific and effective in explaining the reception and understanding of information.

Cultural symbols have multi-dimensional meanings. From the level of consciousness, cognition of cultural symbols is a subjective attitude and emotion (Veissière et al., 2020); from the perspective of behavior, cognition of cultural symbols is a friendly and good interpersonal interaction (Rendell et al., 2011); from the perspective of social relations, cognition of cultural symbols is a key link in maintaining harmony and friendship in social relations (Sinha, 2005). Regardless of the perspectives, it is clear that the emotional elements of cultural symbol cognition are particularly prominent and linked with the phenomenon of empathy. Cognitive empathy and emotional empathy have exerted a progressive effect in the modality generation of cultural symbol cognition, facilitating individuals to develop altruistic and prosocial behaviors (Hoffman, 1977). From the perspective of empathic narrative, empathy is an essential element in the formation of good interactions between different individuals, which is conducive to eliminating cultural discounts and enhancing cultural cognitive feedback (Decety and Lamm, 2006). Empathy is not a feeling, nor an experience, but an ability. Individuals have their own logic and way of thinking. Empathy can only be realized under certain conditions, whether it is positive or negative emotion, asymmetry and distortion inevitably occur in the process of information transmission, and this emotional bias will inevitably affect the individual’s cognition of another individual. Therefore, this study proposes the following hypotheses:

*H1*: The audience feedback has a significant impact on the cognition of cultural symbols.

*H1a*: The emotional empathy has a significant impact on the cognition of cultural symbols.

*H1b*: The cognitive empathy has a significant impact on the cognition of cultural symbols.

*H1c*: The emotional bias has a significant impact on the cognition of cultural symbols.

#### Cultural Symbol Cognition and Cultural Identity

From the perspective of empathic narrative communication, the emotional component in cultural symbol cognition can not only touch the changes of personal inner feelings, but also arouse emotional empathy. Cultural identity restricts itself based on cultural differences and diverse expressions and forms common cultural values through cultural cognition (Sun et al., 2017; Li et al., 2018; Brown et al., 2019; Wang et al., 2021). The cultivation of cultural self-confidence of contemporary young adults in the context of today’s era is a process from cultural symbol cognition to cultural identity, and then to cultural inheritance. It can be seen that cultural symbol cognition has significant influence on cultural identity. But in terms of its present situation, the political culture of contemporary young adults has not achieved the expected effect in the online political communication, the cognition of culture is still far from enough to effectively produce cultural identity. Therefore, this study proposes the following hypothesis:

*H2*: Cultural symbol cognition has a significant impact on cultural identity.

Additionally, the study proposes the following hypotheses on mediating effect:

*H3a*: Cognitive empathy has a significant impact on cultural identity through cultural symbol cognition.

*H3b*: Emotional empathy has a significant impact on cultural identity through cultural symbol cognition.

*H3c*: Emotional bias has a significant impact on cultural identity through cultural symbol cognition.

#### Scale Design and Data Collection

Combining the research conclusions of topic clustering and sentiment analysis obtained by the above-mentioned network text analysis, this study integrated the ACME empathy measurement method to analyze the contemporary young adult’s perception mechanism of political culture in online political communication from the dimensions of cognitive empathy, emotional empathy, and emotional bias. The questionnaire in this study was divided into three parts, which are the individual basic information, the behavioral characteristics, and the cultural cognition of the respondents. Five-level Likert scale was adopted to construct dimension indicators including cognitive empathy, emotional empathy, emotional bias, cultural symbol cognition, and cultural identity, with a total of 19 items in the scale. In line with the ACME model established by Vachon and Lynam (2016), the three indicators of empathy were measured, the participants were asked to recall the experiences of empathy and indicate the degree of recognition (for example, “I make great efforts to absorb the essence of political culture in the online political communication,” “Learning the political culture in online political communication can expand my vision and improve my moral literacy,” “I think the political culture in online political communication is nothing special compared to other cultures,” etc.); besides, pursuant to the scale of cultural symbol cognition developed by Veissière et al. (2020) to measure the degree of contemporary young adults’ cognition of cultural symbols, respondents were asked to think about the influence of cultural symbols on them (for example, “I think that the artistic symbols of the political culture in online political communication demonstrate the wisdoms of the times,” etc.); furthermore, following Brown’s scale to measure the variable of cultural identity, the participants were asked to indicate their degree of identity with the political culture in online political communication (for example, “The publicity and education of political culture in online political communication is inadequate to support my sense of identity and trust,” etc.).

Additionally, based on the setting of the measurement scales in this study, the research participants were composed of the contemporary young adults aged 15–24 in a school. Catering to the requirements of the sample numbers, the Partial Least Squares method (PLS; Wold et al., 2001), capable of processing non-normal distribution data, is selected as the research method. A total of 92 questionnaires were recovered, among which 86 questionnaires were valid, with an effective rate of 93.5%. Moreover, since the PLS-SEM analysis method can effectively prevent the occurrence of the collinearity among the observed variables, the PLS-SEM analysis method can meet the main requirements of this study.

#### Construction and Verification of PLS Structural Equation Model

**Figure 2:**
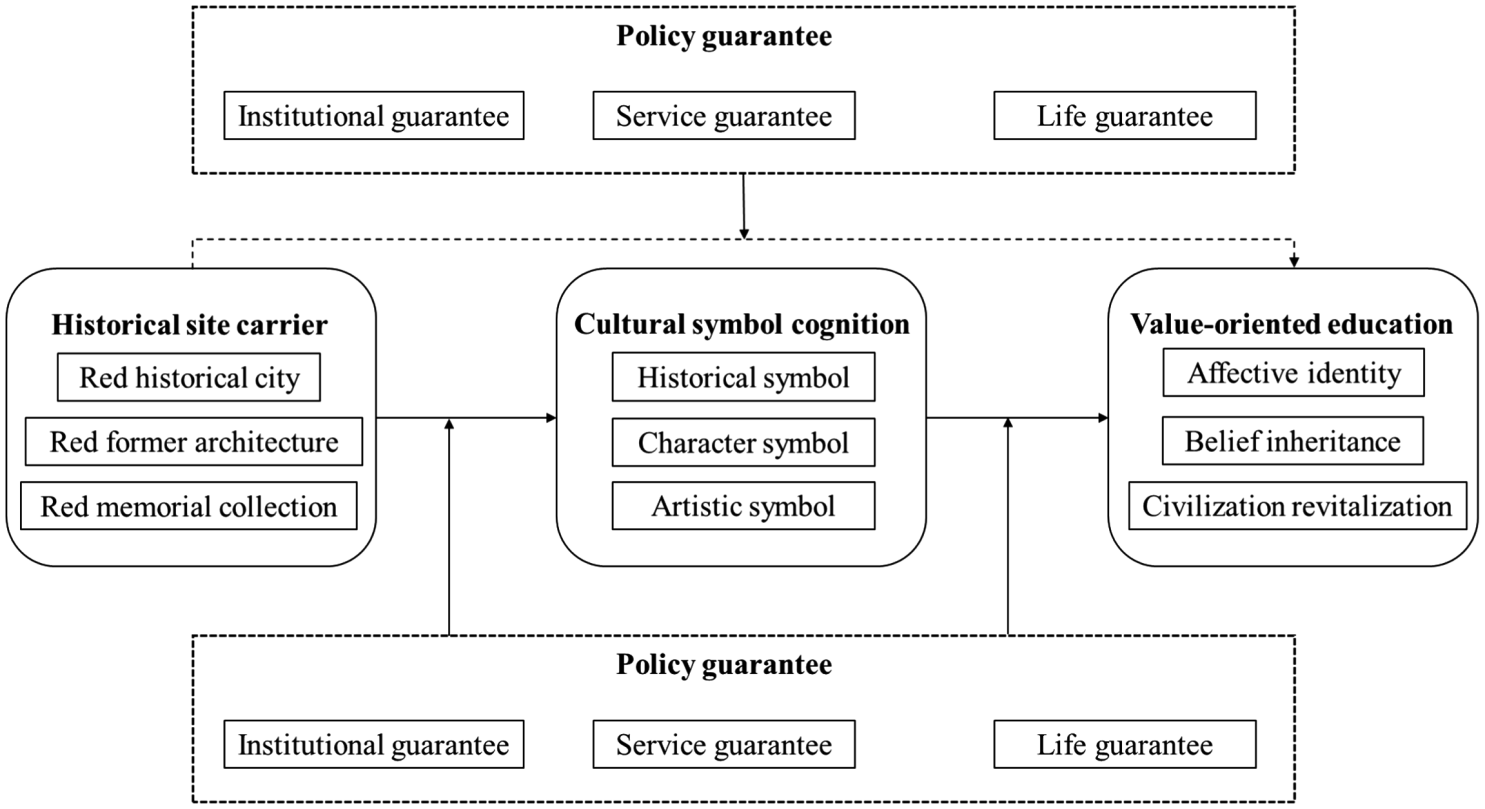
Theoretical model of the political culture communication.

Based on the proposed research hypotheses and ACME scale, the path relation diagram between latent variables and observed variables was constructed. Smart PLS 3.0 software was used to establish the PLS structural equation model of contemporary young adults’ cognition of political culture in online political communication (as shown in Figure 2).

In the PLS-SEM test, Cronbach’s alpha coefficient test results of the five latent variables of cognitive empathy, emotional empathy, emotional bias, cultural symbol cognition and cultural identity were 0.880, 0.918, 0.925, 0.783, and 0.851, respectively, all greater than 0.7, indicating that each latent variable has good reliability; the CR of each latent variable was, respectively, 0.917, 0.942, 0.947, 0.863 and 0.910, which are all greater than 0.7, further indicating that the model has high reliability. The AVE of each latent variable was 0.734, 0.802, 0.816, 0.617, and 0.770 and rho_A was 0.891, 0.922, 0.932, 0.786, and 0.851 respectively, all of which were greater than 0.7. Therefore, the structural equation model of the proposed model passed the fitting index and the test of reliability and validity.

Through the prediction ability test, *R*^2^ was 0.579. Since *R*^2^ was between 0.25 and 0.75, it indicated that each latent variable has a strong explanation for cognition of political culture in online political communication; *Q*^2^ was 0.389, which was greater than 0.35, demonstrating that the exogenous variables of this model have a strong correlation with the prediction of the endogenous variable of political culture in online political communication, and indicating that the PLS model has a strong overall prediction ability regarding the contemporary young adults’ cognition of the political culture in online political communication.

Through the correlation coefficient test, it was found that to construct the correlation coefficient matrix between latent variables (as shown in Table 1), the values below the diagonal value were the correlation coefficients between latent variables respectively, and each latent variable had different connotation in theory and had good discriminant validity.

**Table 1:** Correlation coefficient matrix between latent variables.

	EB	EE	CSC	CI	CE
EB	(0.917)				
EE	0.607	(0.942)			
CSC	0.666	0.646	(0.947)		
CI	0.649	0.575	0.722	(0.863)	
CE	0.607	0.507	0.643	0.793	(0.910)

The value on the diagonal is reliability; and the values below the diagonal are the correlations between variables.

#### Hypotheses Testing

**Figure 3:**
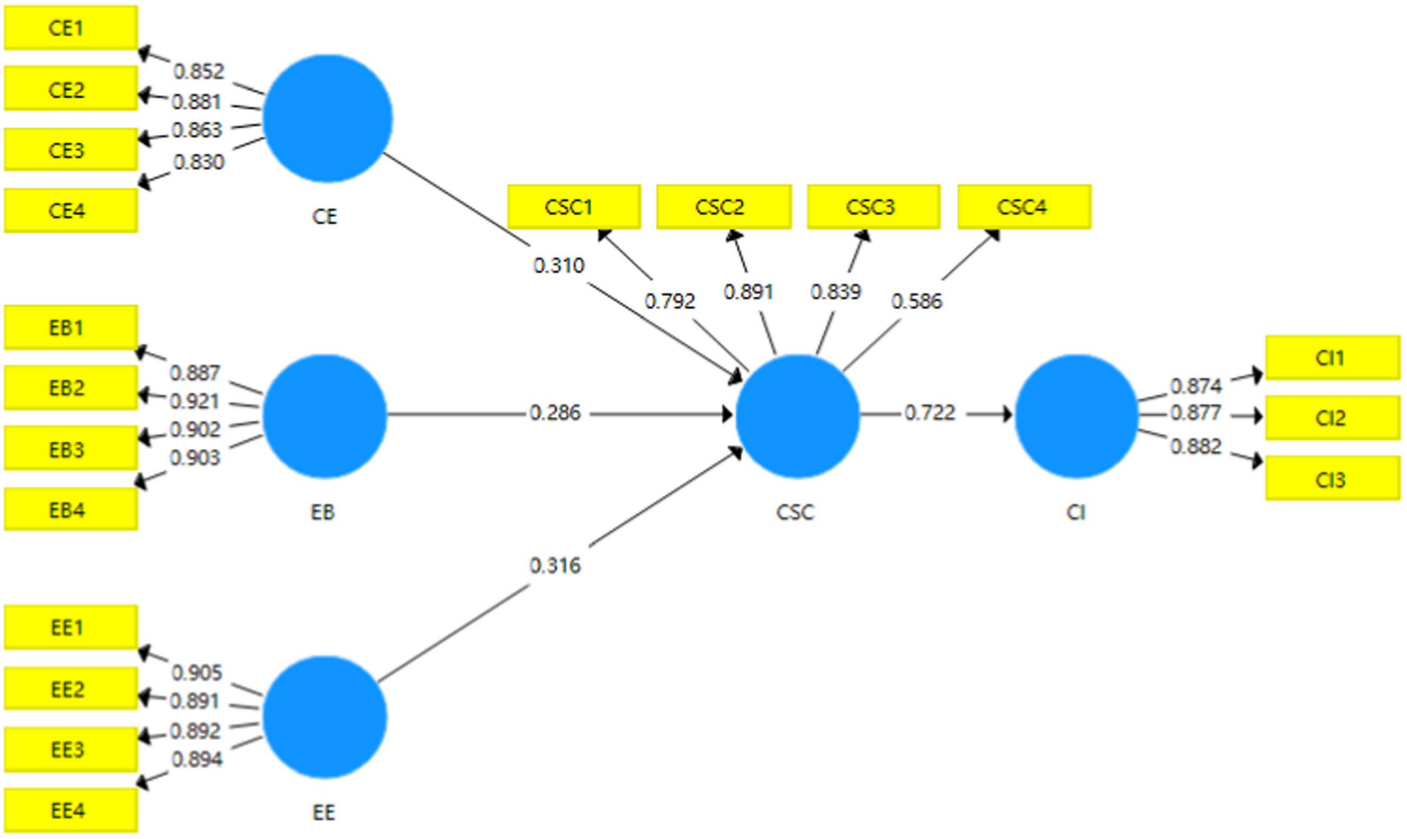
The cognitive path coefficients. CE, Cognitive Empathy; EB, Emotional Bias; EE, Emotional Empathy; CSC, Cultural Symbol Cognition; CI, Cultural Identity.

As shown in Figure 3, the research hypotheses were verified. The results showed that the path coefficient between cognitive empathy and cultural symbol cognition was 0.310, the coefficient between emotional empathy and cultural symbol cognition was 0.316, and the coefficient between emotional bias and cultural symbol cognition was 0.286, and the path coefficient between cultural symbol cognition and cultural identity was 0.722. Generally, the coefficient of each path was basically greater than or close to 0.3, which reasonably and effectively demonstrated the influence path structural model between cognitive empathy, emotional empathy, emotional bias, cultural symbol cognition, and cultural identity, and the significance level p of all paths was less than 0.05; thus, Hypothesis 1, Hypothesis 2, and Hypothesis 3 were all valid.

Furthermore, this study used Bootstrapping to calculate the T statistic of each path coefficient to test the significance level of the path coefficient. There are three mediating effect paths in contemporary young adults’ cognitive model of political culture in online political communication, namely, “cognitive empathy → cultural symbol cognition → cultural identity,” “emotional empathy → cultural symbol cognition → cultural identity,” “emotional bias → cultural symbol cognition → cultural identity,” and the T values in the three mediating effect paths were 2.758, 2.549, and 2.509, respectively, indicating that the three path coefficients had high T statistics and each path coefficient passed the significance testing, which further showed that after repeated sampling, the model structure had high stability and there was a significant indirect effect.

## Conclusion and Implications

### Conclusion

Through the research on the origin, characteristics, and performance of political culture, we can see that the current online political communication has taken on presentations in the context of the times. However, insufficient attention is paid to the feedback of contemporary young adults. Therefore, in accordance with the data analysis and observation of Sina Weibo, it is basically a one-way communication process, that is, in online political communication, there is a lack of feedback on the interaction between the political culture conveyed and contemporary young adults. Such cultural communication mechanism needs to be further improved. This study put forward the extant communication logic in view of the analysis of online texts, and through the questionnaires and empirical analysis, it sorted out the cognitive feedback of contemporary young adults that should be understood at a deeper level.

Specifically, the results of network text analysis demonstrated that the current online political communication followed the logic of “historical site carrier → cultural symbol cognition → value-oriented education” and reflected the managerial and control effects of policy guarantees. However, its value-oriented education could not directly expound that the audience had fully accepted the political culture in online political communication, and its inherent regularities need to be studied through the reverse cognition of the empathy of the audience, thus the Q1 was answered. The empirical analysis results of PLS-SEM showed that the feedback of the audience was mainly carried out in three dimensions including cognitive empathy, emotional empathy, and emotional bias. The audience feedback had a significant impact on cultural symbol cognition, the cultural symbol cognition had a significant impact on cultural identity, and the influence paths and degrees of each variable were different. The audience feedback played a significant role in cultural identity with cultural symbol cognition as a mediating variable, thus the Q2 was answered. Overall, the interaction between online political communication and contemporary young adults can be systematically constructed through empathic narrative direction.

### Research Implications

This study confirmed that the empathic narrative of political culture should become a practical path to improve online political communication, which can be realized through three ways: deepening cognitive empathy, stabilizing emotional empathy, and adjusting emotional bias. Accordingly, the Q3 was answered.

#### Deepening Cognitive Empathy

With a view to better realize the deepening of cognitive empathy, construction efforts can be made to strengthening communication and exchanges and enhancing attention and recognition. In the process of communication and exchange, it is necessary to further understand the feelings and needs of contemporary young adults for political culture in online political communication, and formulate specific and appropriate implementation plans for online political communication so that contemporary young adults are able to recognize and understand the political culture in online political communication in a proactive way; in terms of improving the attention and recognition given to the political culture, the deepening of cognitive empathy capability requires in-depth understanding and accurate grasp of the cultural and ethical needs of contemporary young adults to ensure that the communication contents truly meet the needs of the audience, timely grasping the audience feedback in the process of cultural communication and exchange to make corresponding revisions and adjustments to the communication contents.

However, what is critical at the moment is to actively respond to the concerns of contemporary young adults to answer their questions in a timely manner and eliminate possible doubts and contradictions. Only the communication narratives based on cognitive empathy can be understood and accepted by the majority of young adults. This requires us to follow the universal laws of online political communication and cultural exchanges, build a narrative path that integrates contemporary young adults and political culture in online political communication, and increase the diversity, popularity, and interaction of empathic communication to enhance the objectivity and rationality of the narrative system.

#### Stabilizing Emotional Empathy

In online political communication, guiding and promoting the communication and exchange between contemporary young adults and political culture can facilitate the young adults to view issues from their own perspective and recognize and respect online political communication emotionally and rationally in the hope of enhancing the cultural literacy of contemporary young groups to further realize cultural identity and promote the nurture of cultural confidence. Empathic communication in the digital context can not only be expressed through traditional words, but also through pictures, audio and video means to spread the cultural charms. When outputting cultural products with political cultural genes and emotional factors, local political cultural factors and empathic elements should be integrated into the inheritance and development of excellent culture, and at the same time, the estrangement and strangeness of political culture between contemporary young and online political communication shall be resolved taking account of the current era context to build an effective interactive structure for empathic communication.

Imagination and contact provide a new perspective for people to create fields of emotional empathy. For contemporary young adults who lack the influence and nourishment of political culture in online political communication, imagination and contact can help them simulate how to conduct cultural communication and exchanges and transform what they have learned and thought into emotional empathy. The situational setting that “learning different revolutionary stories of martyrs results in cultural learning” as well as the role simulation of revolutionary heroes can become “experimental projects” for emotional empathy. Such situational simulation allows the majority of young adults to actively participate in it, which is beneficial to enhance their interest and acceptance in a bid to realize the effective interaction.

#### Adjusting Emotional Bias

In the process of analyzing the empathic communication of political culture, the emotional tendencies of contemporary young adults may change, and consequently different emotions will have deviations or conflicts. It is necessary to adjust the emotional bias from the inner emotional level and quantify the cognitive and affective degrees and influences caused by emotional bias in the context of online political communication to lay the foundation for subsequent effective interaction with young adults. Generally, individuals only have positive and negative affective tendencies toward other individuals or time, and it is easy to ignore the problems and contradictions caused by emotional bias among young adults and the impact of their feedback on the empathic communication of political culture. As discussed in this study, with regard to the empathic communication of political culture in online political communication, problems and contradictions are more likely to occur if different young adults have great emotional bias on cultural communication, and the characteristics of empathic narrative will easily intensify such contradictions. As a result, the effective interaction between young adults and political culture in online political communication may be seriously affected, and online political communication may also be notably hindered. Therefore, in the process of empathic narrative of online political communication, we can avail of the connotation of empathy theory, pay attention to young adults’ inner feelings and explore their emotional factors to adjust their emotional bias with an aim to create empathic narrative field and cultivate the young adults’ empathic ability so that the empathic narrative path of online political communication will become the endogenous driving force for deepening the effective interaction between contemporary young adults and political culture in online political communication.

## Data Availability Statement

The original contributions presented in the study are included in the article/supplementary material, further inquiries can be directed to the corresponding authors.

## Ethics Statement

The studies involving human participants were reviewed and approved by Jiangxi University of Finance and Economics. Written informed consent for participation was not required for this study in accordance with the national legislation and the institutional requirements.

## Author Contributions

YW and LL contributed equally to this work and they contributed to the empirical work and the analysis of the results. LL advised the hypotheses development and revised manuscripts. ZZ and JZ supported the total work of the YW. LL was in charge of the article writing, communicating with the reviewers, and made multiple revisions for final publication. All authors contributed to the article and approved the submitted version.

## Funding

This study is supported by funding from the Project of National Social Science Foundation of China (No. 15BXW052), and from the Jiangxi Social Science Foundation Youth Project, (No. 21JY54), “Research on the affective bias and guidance of Jiangxi university students’ network cluster behavior from the perspective of patriotism education.

## Conflict of Interest

The authors declare that the research was conducted in the absence of any commercial or financial relationships that could be construed as a potential conflict of interest.

## Publisher’s Note

All claims expressed in this article are solely those of the authors and do not necessarily represent those of their affiliated organizations, or those of the publisher, the editors and the reviewers. Any product that may be evaluated in this article, or claim that may be made by its manufacturer, is not guaranteed or endorsed by the publisher.
